# Development of a recycling machine for constructing synthetic yarn from plastic waste

**DOI:** 10.1016/j.mex.2024.102749

**Published:** 2024-05-05

**Authors:** Nuzat Nuary Alam, Md. Mehrab Sadik, Tahmid Shahriar Arnob, Isfak Habib Iftu, Abrar Jahin Khan, Kazi Firoz Ahmed, Rethwan Faiz

**Affiliations:** American International University-Bangladesh, Dhaka, Bangladesh

**Keywords:** PET recycle, Extrusion, Eco-friendly textile, PET bottle, R-pet, Sustainable textile, Plastic extrusion, Extrusion of plastic thread from plastic waste

## Abstract

A major challenge in plastic recycling is to convert plastic waste into a useful product. For this transformation, sustainable technologies such as plastic recycling machines are required. Current technological concepts of plastic recycling are fairly similar. This study aims to develop a small and economical plastic recycling machine to enhance microenterprise by supplying simple equipment for recycling locally processed plastic waste into thread. Starting with a hopper at the input end, the machine incorporates an auger inside a barrel, which is then linked to a metallic perforated mold at the output end. With the help of the system, the plastic flakes melting process is facilitated by maintaining temperatures between 170 °C to 211 °C at equispaced locations of a uniform barrel, while the auger spin enables the flow of molten plastic forward towards the mold.The mold reshapes the liquid plastic into strings of thread. The machine exhibits higher efficiency, reaching approximately 75 % at a decreased screw speed, as low as 28 rpm. It also achieves an average throughput of 156 gm/hour at the lowest specific mechanical energy (SME) consumption. The prototype consumes 1.5 kW for an hour operation. The entire system requires minimal space, making it appealing to individuals with limited financial resources to start a new business venture.

• A sustainable technology to recycle plastic waste into plastic thread.
• This optimized, portable and robust system ensures safety and lower operating costs.
• This system does not require prior knowledge for operation, hence encouraging small entrepreneurs.

## Graphical abstract

**Figure:**
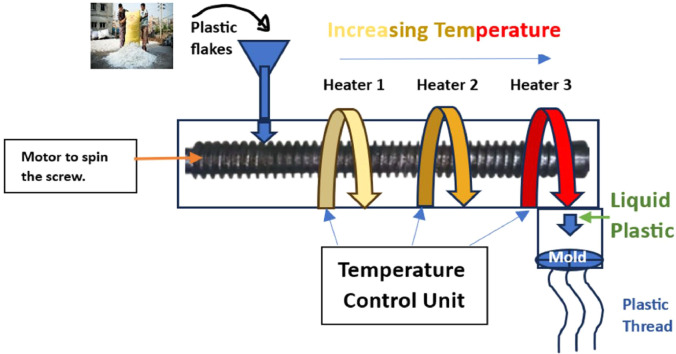

Specifications table

**Table:** 

Subject area:	Engineering
More specific subject area:	*Plastic waste recycling machine.*
Name of your method:	*Extrusion of plastic thread from plastic waste.*
Name and reference of original method:	N/A
Resource availability:	Resources can be obtained from the corresponding author upon request.

## Method details

### Background

Plastic when invented, was an amazing invention that helped the general people to live their daily lives more easily. But soon, this wonder of science became a threat to nature as it is nonbiodegradable. The diverse and multilayered impacts of plastic create significantconcerns for both biological health and environment. These impacts includ agricultural farmlands, groundwater quality, marine and land ecosystems, food toxicity and human health hazards [1,2]. Polyethylene terephthalate (PET) is one of the largest sources of plastic waste and is produced globally each year to make single-use beverage bottles, packaging, clothing, and carpets. Moreover, PET bottle waste is one of the most promising sources for elaborating on waste-to-treasure concepts [3]. The two most widely used technologies for PET waste management are landfilling and incineration [4,5], but both methods have negative environmental impacts such as producing volatile organic compounds [5] and releasing toxic gases and carbon dioxide.. Hence, recycling becomes one of the most common and effective ways and contributes to PET plastic waste management. By selecting a suitable technology [6] for recycling, PET bottle waste can be transferred from an open-loop process to a closed-loop one. Several studies on PET recycling methods have suggested that mechanical recycling currently appears to be the most desirable industrially ubiquitous technique for the management of PET waste, as compared to chemical recycling and incineration [7]. In, this technique PET waste is subjected to mechanical processes including shredding, grinding, washing and/or drying and melting [8].

An extruder machine is used in mechanical recycling to melt plastic flakes. This mechanism uses heat and rotating screws to induce thermal softening or plasticization inside temperature-controlled barrel for producing fixed cross-section extrudate. Dave Hakkens and his team were the precursors to design and create a simple extrusion machine for plastic recycling [9]. In recent times research is going on high-quality precision extrusion recycling technologies such as twin screw [10] and multi-screw [11] extruders, automatic sorting technology [12], efficient cleaning and melting equipment [13], developing “bottle to bottle” technique [14], and made a great achievement. As a result, a good number of state-of-art plastic extrusion machines are commercially available, but both the cost and electricity consumption of those models are quite high. The project is targeting stakeholders with lower purchasing capacity hence, simple structure and minimally complex extruder can make the machine economical. This study focuses on single screw extrusion, a prominent technique in plastic recycling and product manufacturing. Research in this area has yielded diverse approaches and findings. One study utilized a single screw extruder with a single temperature zone to melt plastic, finding optimal production at 190 °C and plastic liquefaction at 230 °C [15]. In contrast, another study employed a single screw extruder with three temperature zones for melting process, requiring a maximum temperature of 260 °C for plastic liquefaction [16]. Additionally a different study employed a simulation based single screw extruder with multiple temperature zones, featuring a unique setup where the screw remained stationary while the barrel rotated; plastic liquefied at a maximum temperature of 180 °C [17]. Similarly, another study used a single screw extruder, with plastic liquefying at a maximum temperature of 250 °C [18]. In these studies the motor speeds varied between 30 rpm and 110 rpm.These literatures showed that in single screw extrusion mechanism selection of parameters mostly the melting temperature andscrew rotation speed, significantly influence the production of an optimal product, including aspects such as mold filling, shape, and appearance.. There are plenty of products that a single screw extruder machine can create from plastic waste such as brick [19], filaments [15], raw material for 3D printer [13,18], synthetic fiber [16,20], construction materials [21], plastic tube [17], and etc. In this paper implementation of a single screw extruder machine to recycle plastic into yarn has been presented which is cost effective, consumes low power, and requires simple operation. The temperature control for melting process has been examined semi-physically using Simulink to identify the temperature range for the actual time operation.

## Design and construction of the plastic recycling machine

The main function of this machine is to heat the plastic to become thread extruded. In this process plastic is gradually melted by both mechanical and thermal energies generated by screw rotation and heaters, respectively.

### System architecture

**Fig. 1:**
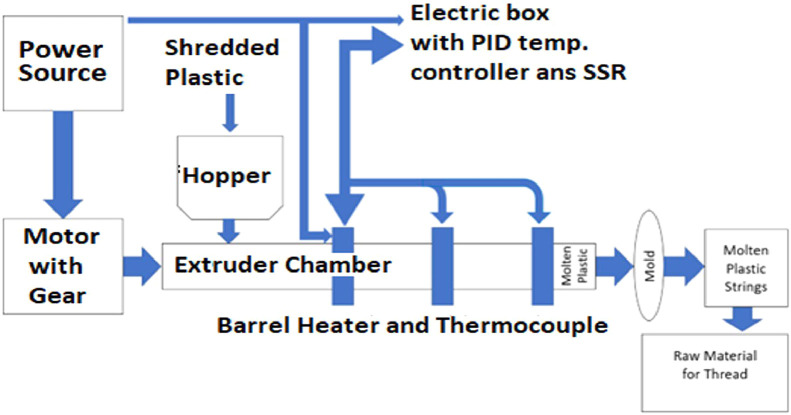
Basic block diagram of the proposed model.

The system has two parts, mechanical and electrical. A block diagram of the system is shown in Fig. 1. The mechanical part comprises an extrusion chamber, barrel, and the nozzle. One end of the barrel is denoted as the hopper or input portion. On the opposite end, a nozzle is attached, which is the output portion. The nozzle incorporates a custom mold to create the plastic strings. The shredded plastic is inserted through a cone hopper, which goes directly inside the screw chamber.

The electric part controls the heating mechanism and the geared motor mechanism. A motor attached to the screw facilitates its turning within the barrel chamber. The rotation speed of the chamber is controlled by the geared mechanism of the motor. The barrel is divided into three different sections: (i) hopper end, (ii) barrel body and (iii)nozzle end.

The separate heater is attached to the outer surface of each section of the barrel to create separate temperature zones. Thermocouples are connected on the barrel next to the barrel heaters to monitor the temperature of each heater. The temperature gradually increases from hopper end to nozzle end. Temperature of the heaters was regulated by the PID temperature controller. The controller gets the temperature reading from thermocouples. Indirectly, the motor is linked to the screw via a pulley to prevent the barrel's temperature from impacting the geared mechanism.

**Fig. 2:**
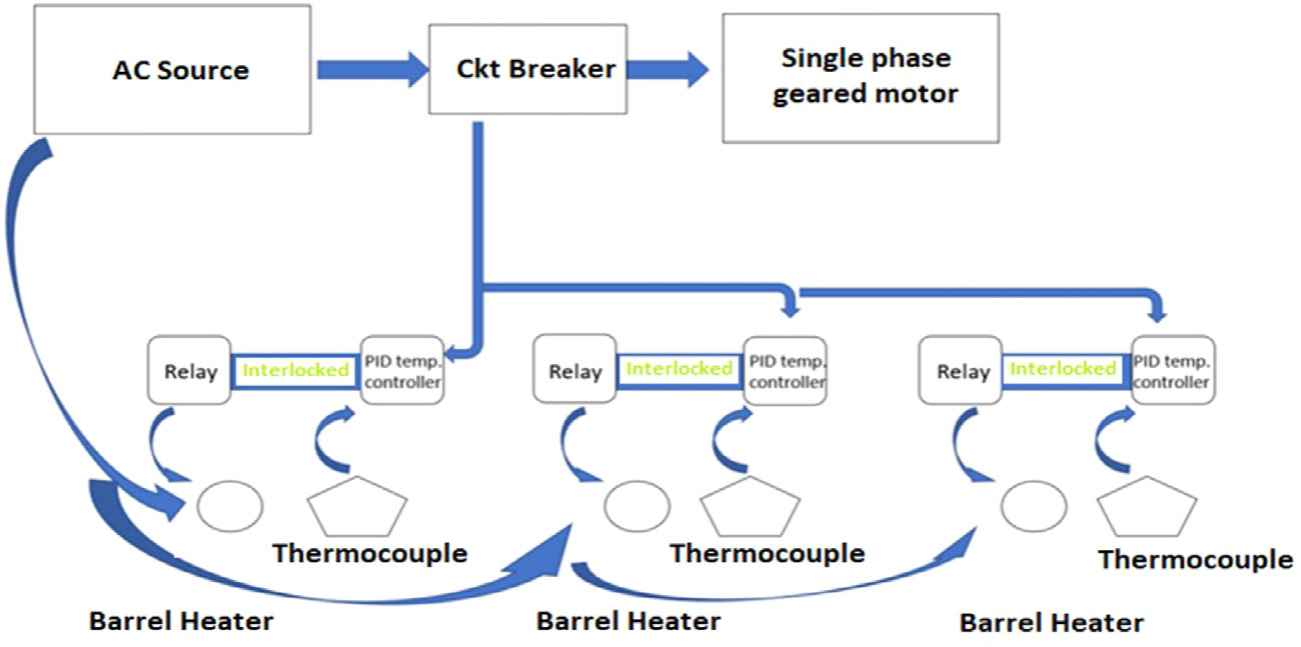
Block diagram of the electrical model.

Fig. 2 shows the block diagram of the electrical system where an AC source powers the motor and PID controllers through a circuit breaker for protection. It also powers the heaters integrated with thermocouples. The controllers are connected to the heater through relays. Thermocouples read the temperature of the heater and inform the PID to execute the switching operation.

**Fig. 3:**
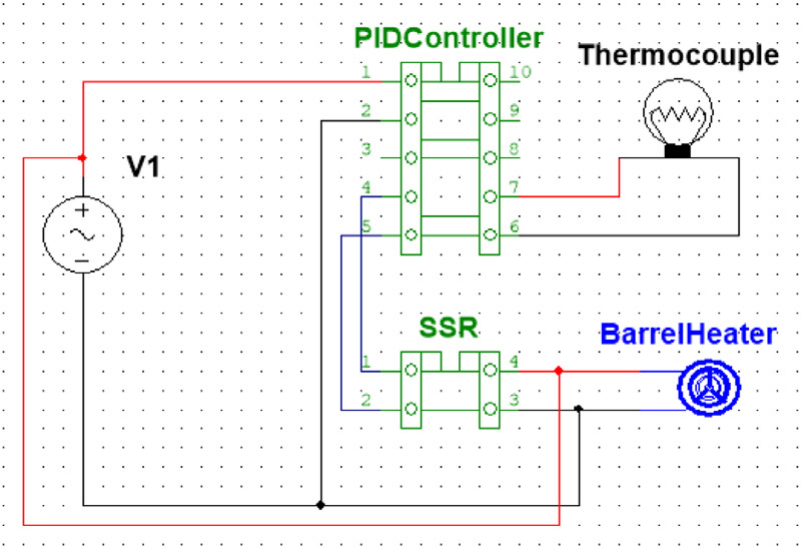
Schematic of the temperature unit.

**Fig. 4:**
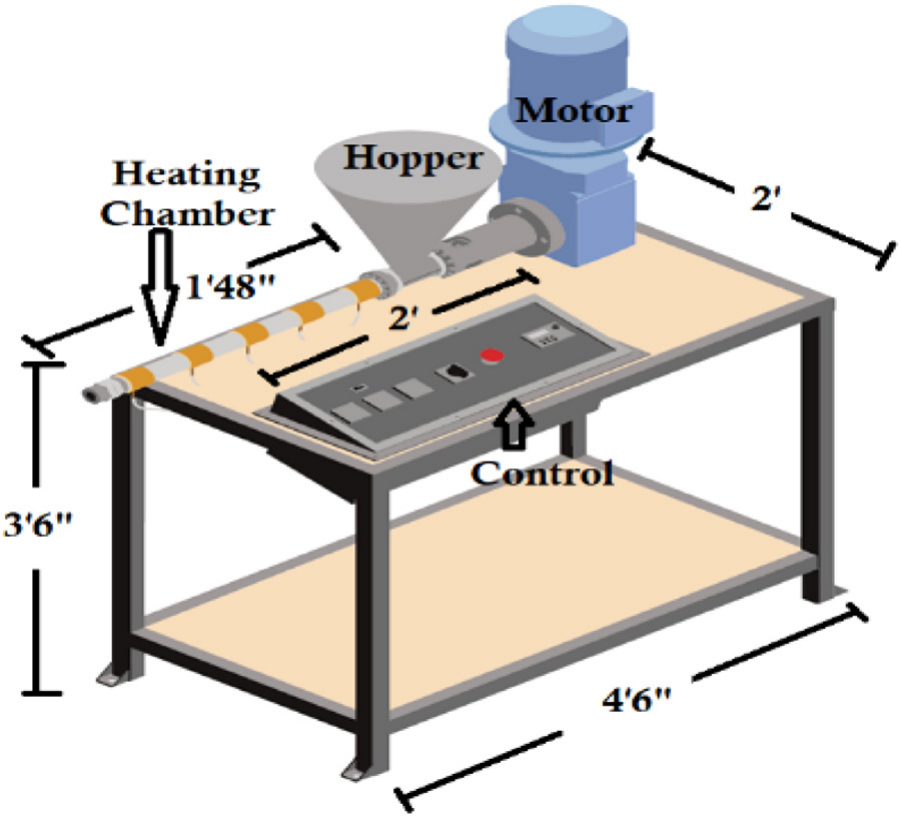
3D model of the proposed machine.

The relay turns on when the temperature of heaters goes down to the desired value, and it turns off when temperature of the heaters exceeds the desired value. A schematic of the temperature control unit is shown in Fig. 3. A 3D model is shown in Fig. 4 where the dimension specification of the machine is also presented.

### Simulink model of the electrical system

**Fig. 5:**
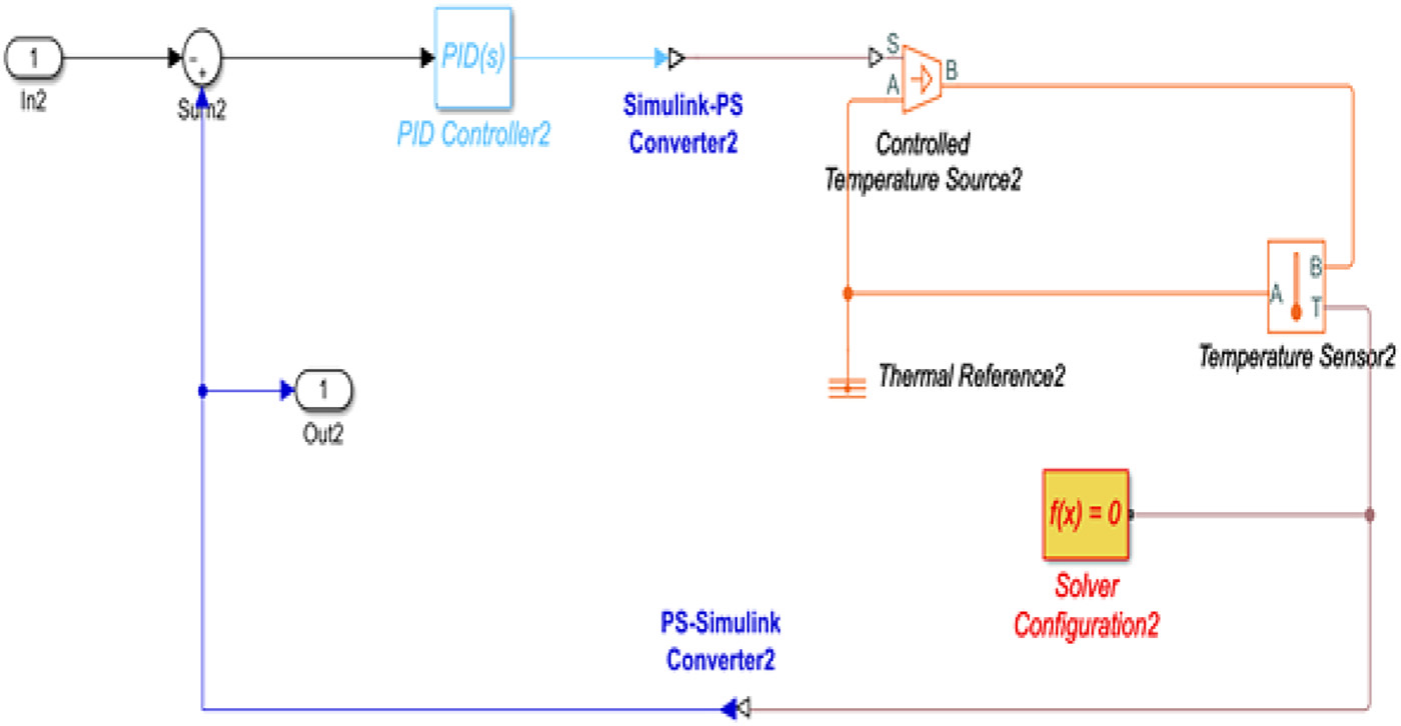
Simulink Model of Temperature Control Unit.

The Simulink model of the temperature control unit for the project was done in Simulink simulation tool of MATLAB R2020a. Fig. 5 shows a detailed model of the temperature control unit.

**Fig. 6:**
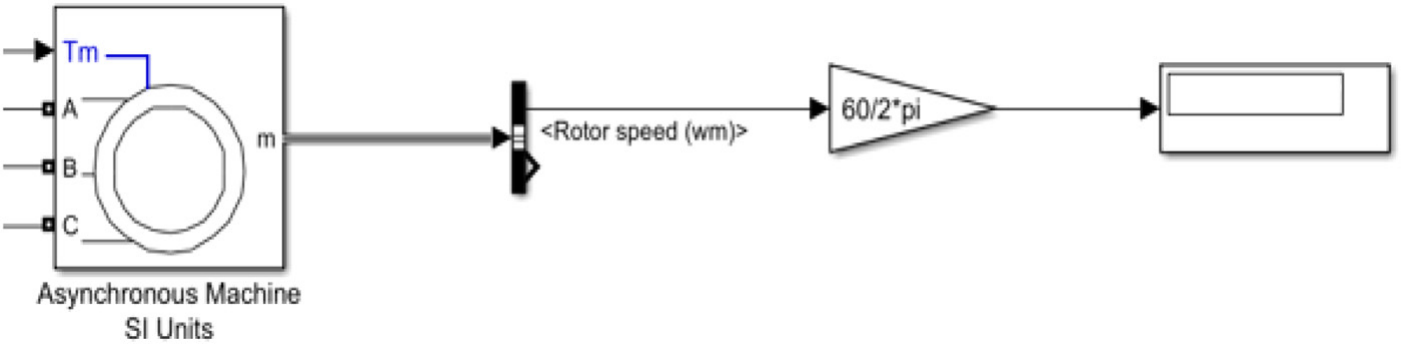
Simulink Model of motor circuit.

Here, the PID controller controls the temperature with the help of a temperature sensor. In the complete temperature control system contains three units, such as this. The simulation model of the motor circuit is shown in Fig. 6. Here using Simulink equivalent circuit parameters of the three-phase induction motor were used in the simulation and understand the variability of the rotor speed.

**Fig. 7:**
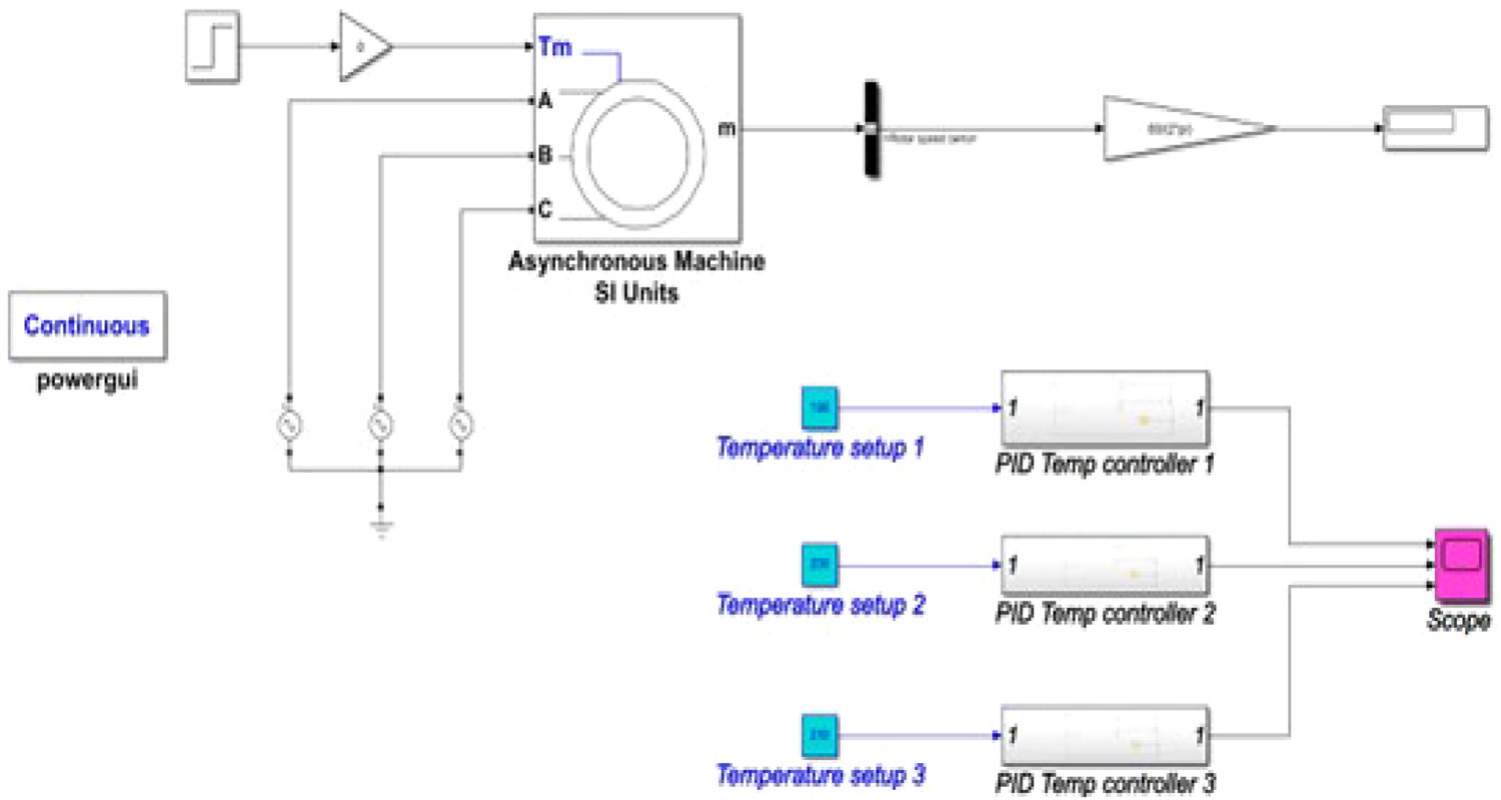
Simulink Model of the electrical unit.

The Simulink model of the complete temperature control system is shown in Fig. 7.

## Hardware implementation

The complete machine is implemented by dividing the work in two steps, the mechanical part and the electrical part. The mechanical part of the project is constructed with intensive and advanced lathe and milling work which was done in a local lathe workshop and the electrical part was compiled in the laboratory. Table 1 shows the list of the components to implement this machine.

**Table 1:** Component list, cost and sources of components.

Serial	Component Name and Description	Model specification	Unit	Per unit price	Total price	Source
1	Geared Motor 1-phase AC 61 RPM	Germana VIEM (1.5 HP, 50 Hz)	01	$92/motor	$92	https://habibmotors.com/
2	Extrusion Input	Cone hopper	01	$5/hopper	$5	http://surl.li/qnlek
3	Extrusion Chamber	Barrel and screw	01	$55/barrel and screw	$55	http://surl.li/qnlek
4	Extrusion Output	Iron mold	01	$9/mold	$9	http://surl.li/qnlek
5	Heating element	Barrel Heater (2 band Construction) 40mm*42 mm	03	$4/heater	$12	http://surl.li/qnlkv
6	Temperature Controller	PID Model REX C-100	03	$14/controller	$41	https://robotechshop.com/
7	Relay	Solid State Relay (SSR4 20A)	3	$11/relay	$33	https://store.roboticsbd.com/
8	Temperature sensor	Clamp thermocouple with module	3	$14/sensor	$41	https://store.roboticsbd.com/
9	Wires	220 V wires	0.5 coil	$5/coil	$2	http://surl.li/qnlzy
10	Motor and Extruder Connector	Pulley	2	$5/pulley	$10	http://surl.li/qnlek
11	Workbench	Supporting table	1	$28/table	$28	http://surl.li/qnlek
				Total Cost	$328/ Unit	

Table 1 delineates the cost breakdown, detailing each component necessary for the production process of the recycling device. The table specifies the name of each component, the quantity required, the cost per unit, the total cost, and the source of the components. This detailed breakdown is essential for accurate cost estimation and budgeting purposes. The cumulative cost of all individual items totals $328. This comprehensive overview provides insight into the overall expenses associated with the production process.

### Step 1: Construction of the mechanical part

The barrel, screw, hopper, and mold are the main parts that were constructed using lathe works. The pulley that is required to rotate the screw inside the barrel is also a part of the mechanical side.

**Fig. 8:**
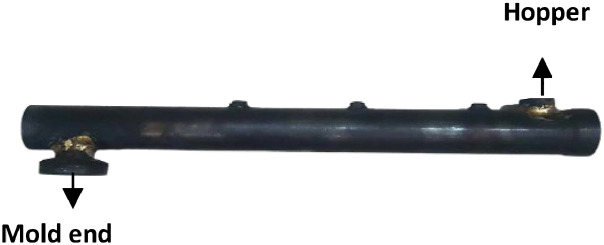
Constructed barrel.

Fig. 8 shows the barrel that holds the screw inside. On the leftmost side, a cut is made to insert the hopper. And on the other end, a portion was created to hold the mold. A ball bearing can be inserted on the furthest right side of the barrel to support the screw within the chamber, therefore minimizing friction.It also has three small screws on top to keep the thermocouples in place. The specification of barrel is shown in Table 2.

**Table 2:** Barrel specification.

Barrel Specification	Dimension in cm
Length	45
Diameter	4
Distance between Thermocouple Clamps	9.5
Mold diameter	4.5

**Fig. 9:**
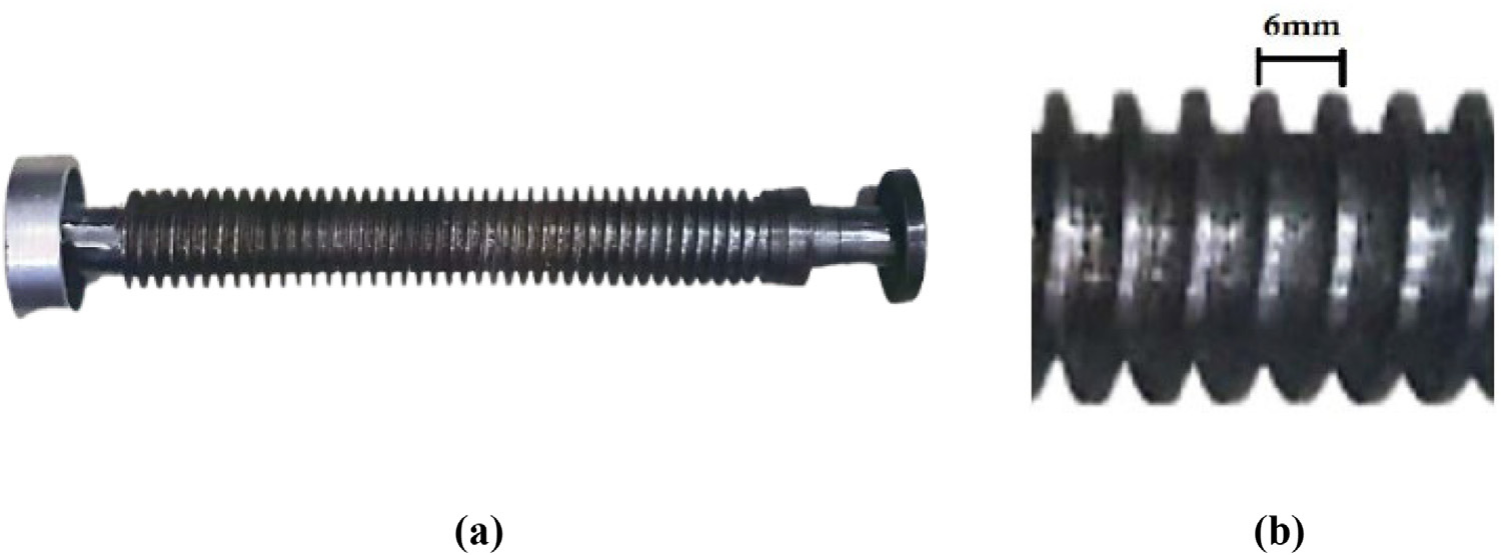
(a) Constructed screw and (b) teeth distance.

Fig. 9 shows the screw that pushes the plastic particles inside the chamber and the specification of screw is given in Table 3. The tooth of the screw has distance of 6 mm between each other. Both end of the screw has ball bearings to reduce friction inside the chamber.

**Table 3:** Screw specification.

Screw Specification	Dimension in cm
Length including shaft	47
Shaft length	9.5
Teeth height	0.5
Teeth distance	0.6

**Fig. 10:**
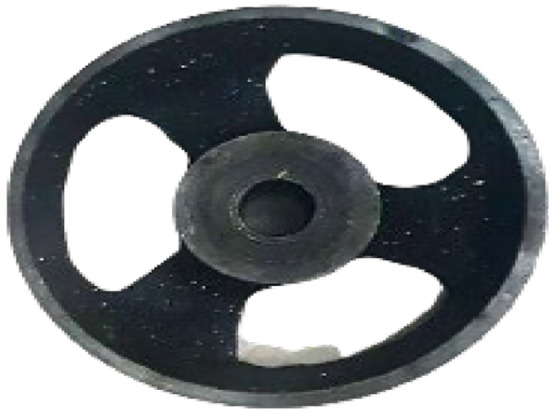
Constructed pully.

The pulley gets connected at the left end of the screw and it is also linked to the motor through a belt. Pully rotates the screw inside the barrel at the speed of the motor speed. The construction of pully is shown in Fig. 10.

**Fig. 11:**
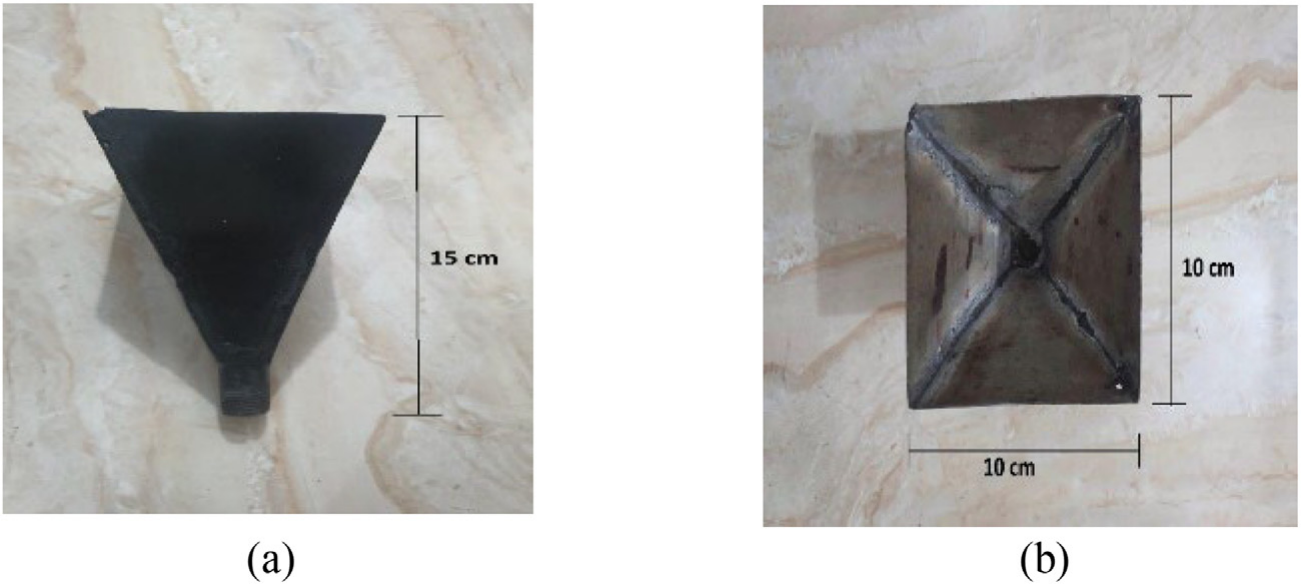
Constructed cone hopper; (a) side view and (b) top view.

The cone hopper is constructed in such a way that it can be removed from the barrel at any time. Fig. 11 shows the constructed hopper.

**Fig. 12:**
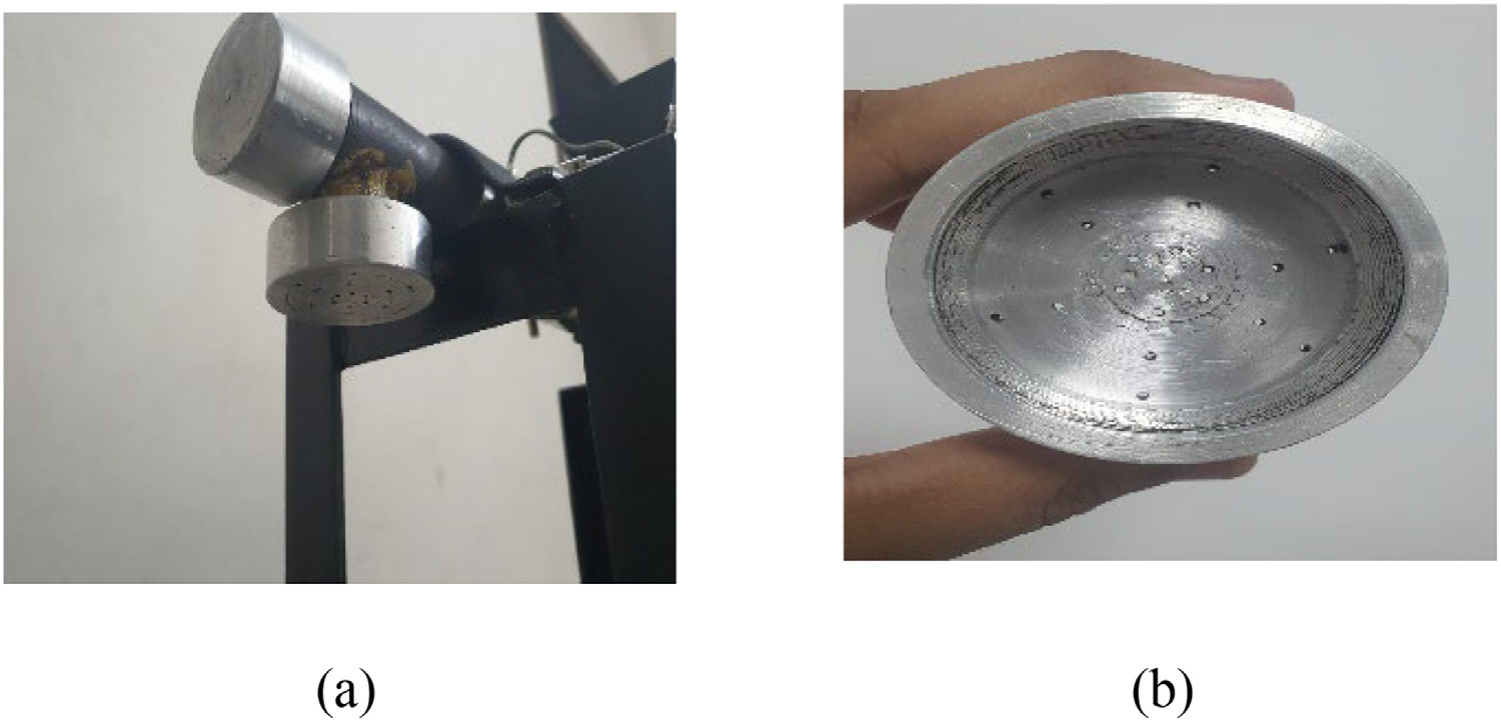
Mold construction (a) mold attachment and (b) holes distributed across the area.

The mold, which shapesthe synthetic string output, features tiny holes spread across its entire surface. Fig. 12 shows the mold attachment alongside the barrel output end, as well as the surface design of the mold.

### Step 2: Construction of the electrical part

The electrical components comprises the motor, thermocouples, barrel heaters, and the electrical box that has PID temperature controllers, SSR4, and connection wires inside.

**Fig. 13:**
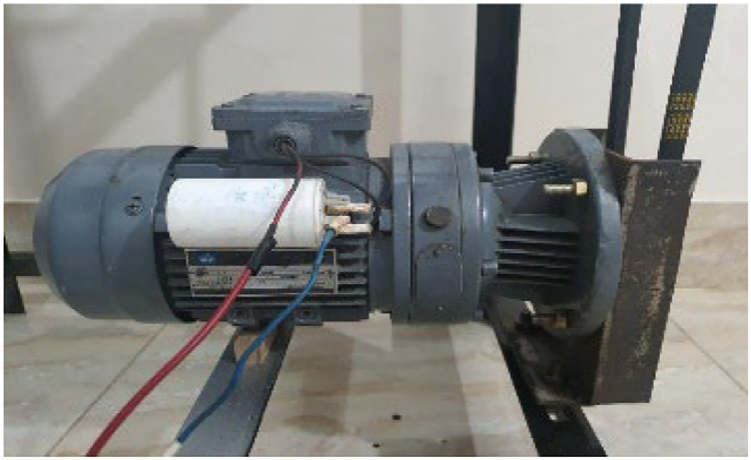
1.5 horsepower (HP) geared motor.

Fig. 13 shows the motor that is used in the prototype. It is a geared motor which has a geared mechanism compiled inside by the factory. Placing a pulley at the shaft to connect the belt was the only modification made in motor hardware. The specification of the motor is shown in Table 4.

**Table 4:** Motor specification.

Motor electric parameters	Value
Voltage	220 V/380V
Power	1.1kW
Speed	61 rpm (rotation per minute)
Mold Connection	Δ/Y

**Fig. 14:**
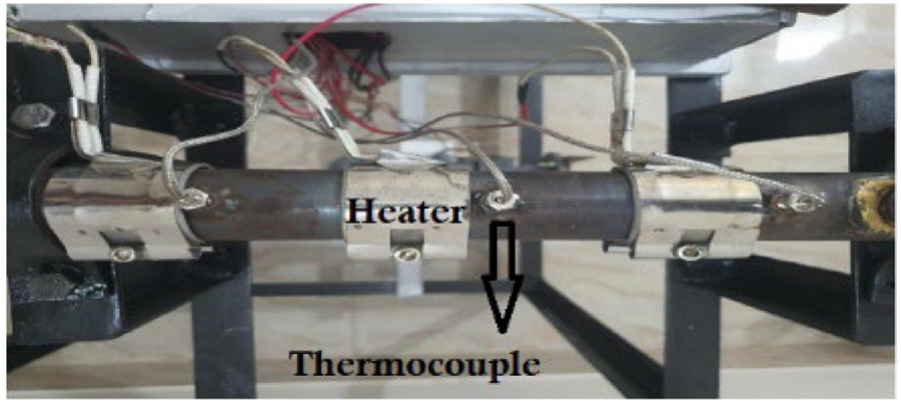
Heater and thermocouple connection.

A geared motor has been used in this design as low rpm (rotation per minutes) was require at high torque to rotate the screw. Hence plastic could travel towards the nozzle at a low speed which facilitates the melting process. Fig. 14 shows the connection of the barrel heater and the thermocouples. The heater and thermocouples are placed very close to secure the accurate temperature values. The sensitivity of the thermocouple is −60 °C to 400 °C.

**Fig. 15:**
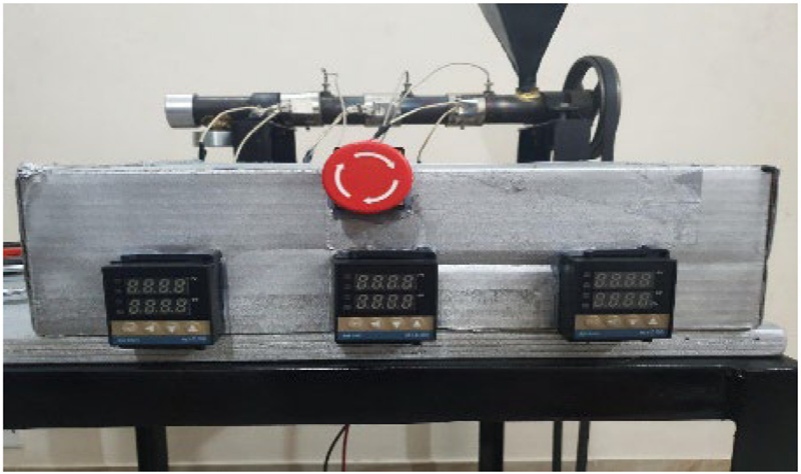
PID controller screens and emergency switch.

The three screens of the three PID temperature controllers are shown in Fig. 15. The electric box also has an emergency stop switch to stop the entire mechanism at any time.

**Fig. 16:**
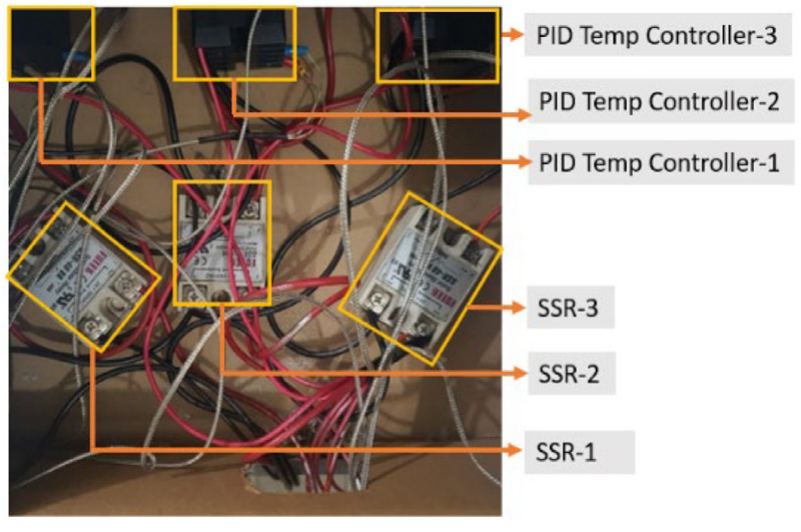
Electrical wiring of the temperature control unit includes red wires for phase connections, black wires for neutral connections, and gray wire for thermocouple connections.

**Fig. 17:**
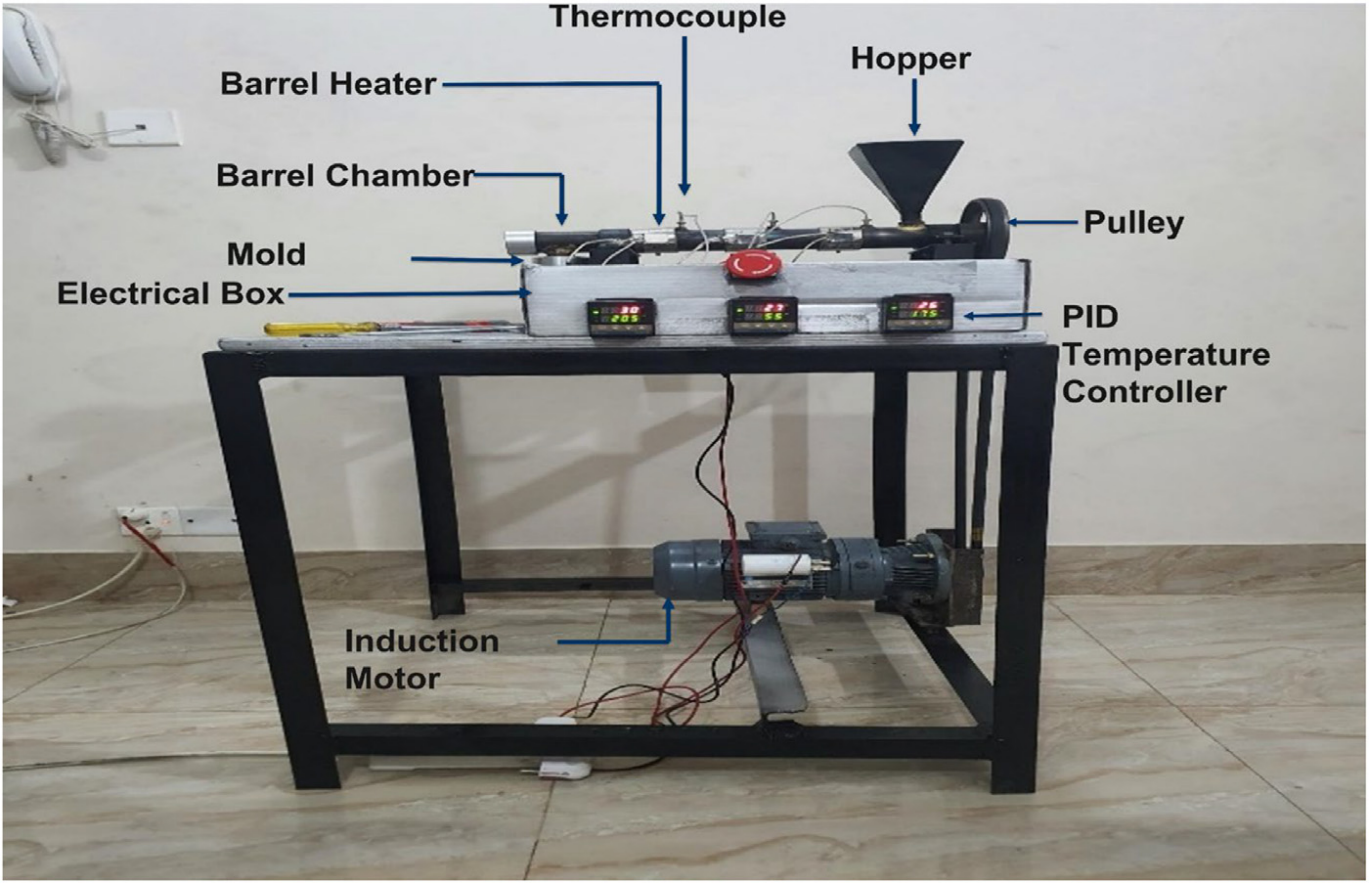
Complete hardware of the proposed model.

Fig. 16 shows the inside of the electric box which holds three SSR4 for three PID temperature controllers. It also has all the wiring connections inside. The wires are connected by following the circuit schematic diagram of the electrical box shown in Fig. 3. The full set of project hardware is shown in Fig. 17, with each component labeled accordingly.

### Step 3: Experimental setup

The experimental setup is shown in Fig. 17 demonstrates the process wherein the plastic flakes were introduced into the hopper and directed into the barrel. The screw inside the barrel transports the flakes towards the mold. Throughout this process, the flakes undergo a temperature increase, gradually rising from the hopper end to the mold end. Consequently, the flakes liquefy, and a string is extruded through the mold.

## Method validation

**Fig. 18:**
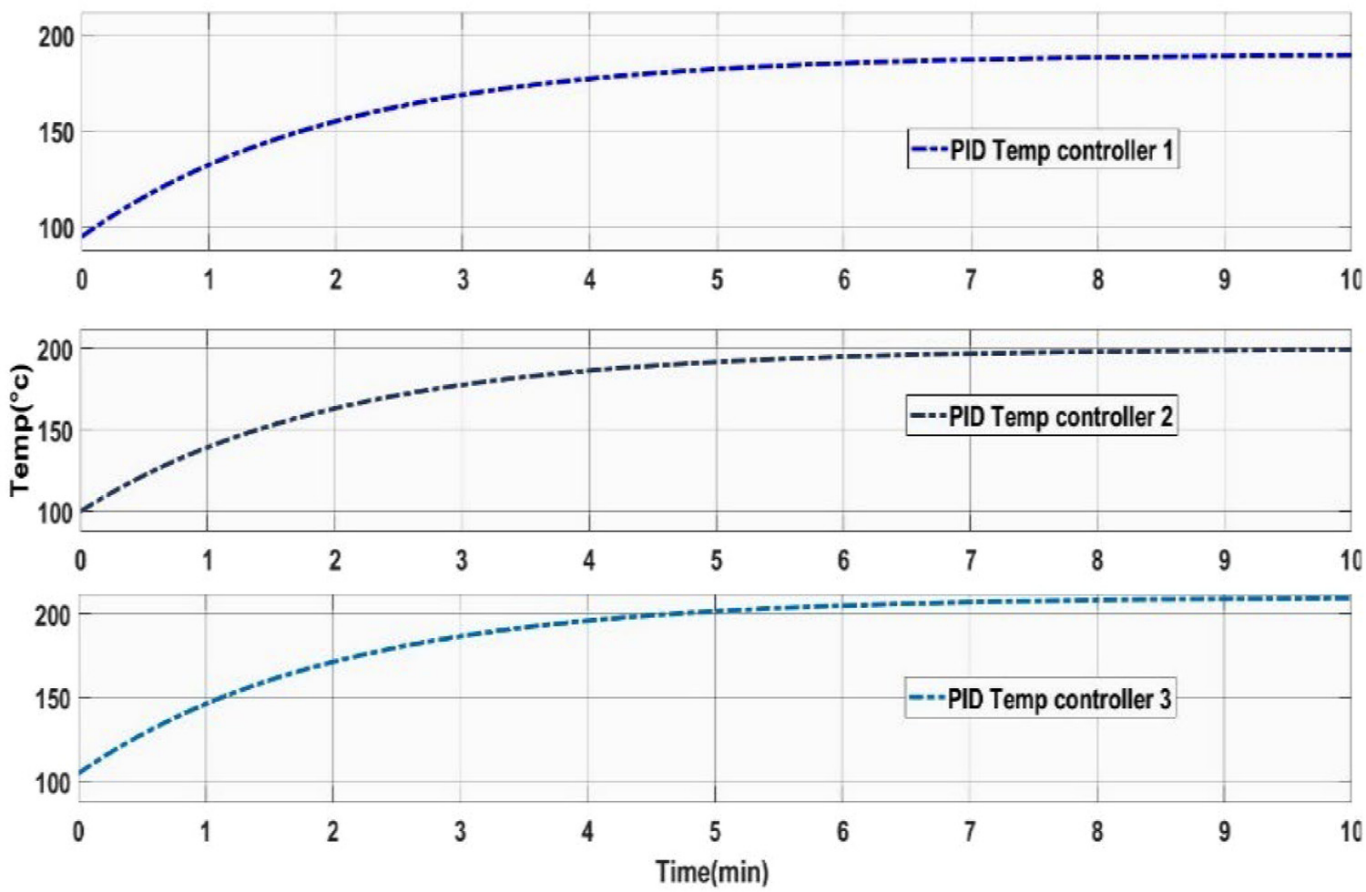
Simulation outcome of the temperature control unit.

The Simulation outcome of the temperature circuit is shown in Fig. 18. Based on the literature, a heating profile has been established, consisting of a temperature range between 230 °C to 250 °C [20]. This range ensures the gradual melting of plastic pellets as they are fed through the barrel, preventing polymer degradation caused by overheating [20]. Within this setup, the barrel comprises three heater zones, accumulating the temperature from a low of 170 °C to a high of 211 °C. This gradual rise helps alleviate the potential for overheating, which might otherwise result in polymer degradation.

**Fig. 19:**
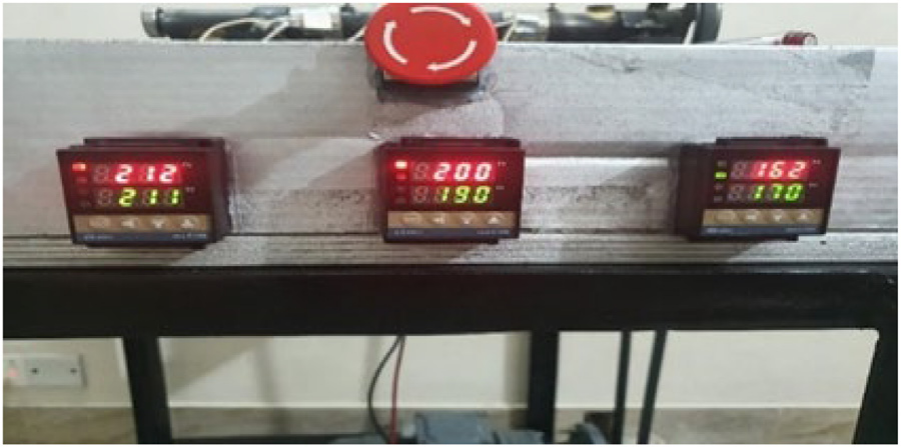
Complete hardware of the proposed model.

In Fig. 19, the output of the hardware is displayed. The green reading signifies the desired temperature, while the red reading corresponds to the temperature detected by the thermocouples, highlighting the difference between the two. The desired temperature and the thermocouple reading is very close.

**Fig. 20:**
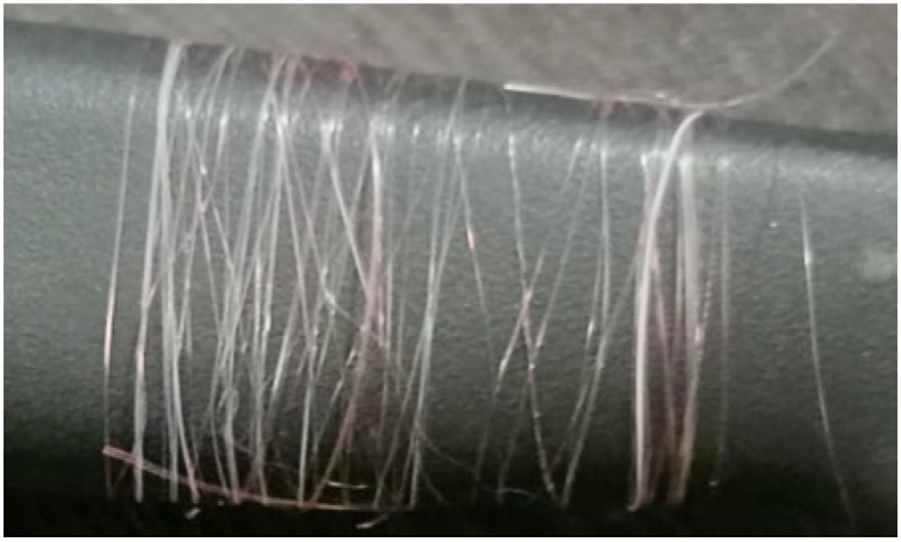
Thread sample.

When the threads came out through the mold those were collected into a trough containing cold water. Then the threads tenderly were guided into a characteristic twisting. The final output of the system is shown in Fig. 20. The amount of thread is shown in the Fig. 20 is obtained in approximately 15minutes.

Extruder screw speed was kept low to avoid the frictional heat generation, otherwise the residence time of the plastic would be too short to prepare a uniform, well-mixed melt. In this model screw runs more slowly, as low as approximately 30 rpm which generate less heat for equivalent mixing. This guarantees that process temperatures remain low and maintains the thermal history, ultimately reducing the requirement for stabilizers.

**Fig. 21:**
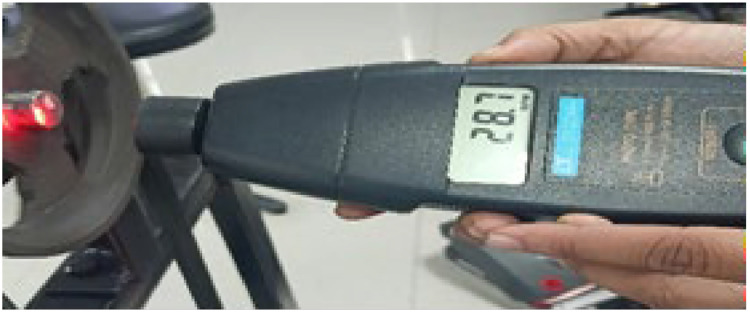
Hand tachometer reading.

The rotation of the motor is measured with the help of a hand tachometer and is shown in Fig. 21. The shaft speed of the induction motor was measured at 61 rpm. However, the translation of this speed to the screw occurs when it is connected to the motor through pulleys.

**Fig. 22:**
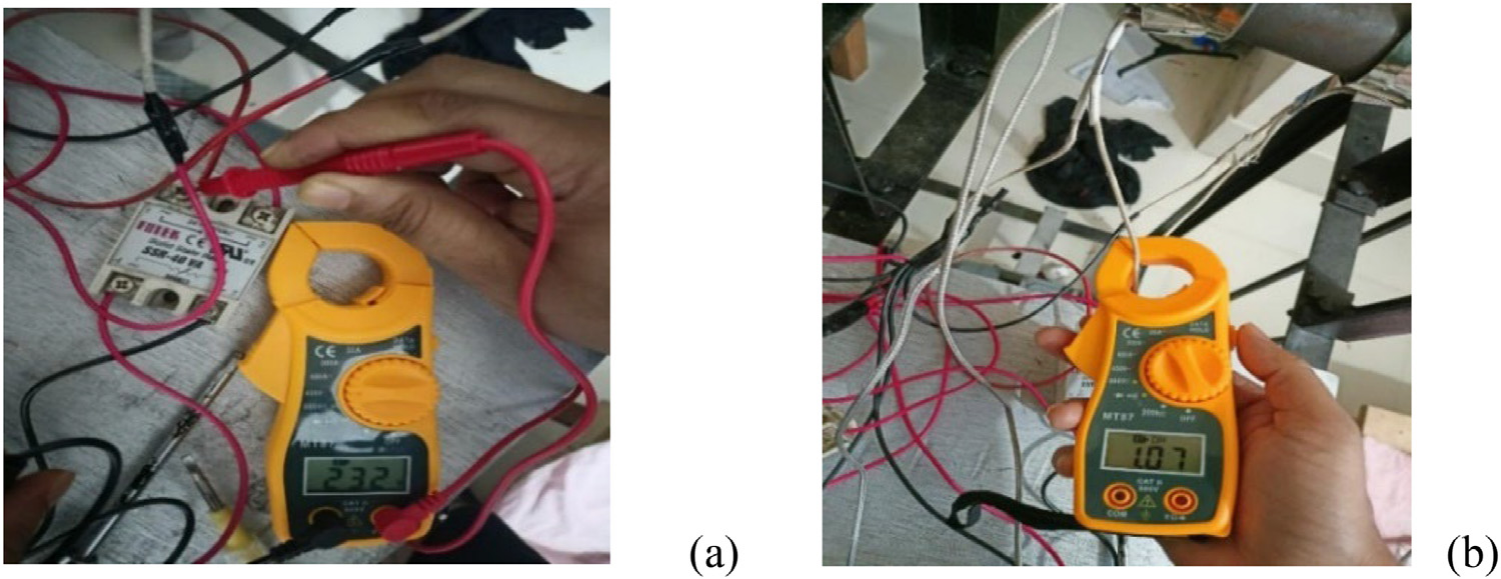
Barrel heater electric parameter measurement. (a) Voltage reading and (b) Current reading.

The setup for the data collection from the barrel heater is shown in Fig. 22. With the help of the clamp multimeter, voltages, currents and other electric parameters were recorded in Table 5. The total electric energy consumed by the heating unit is 0.364kWhr.

**Table 5:** Data collected for barrel heaters.

Barrel Number	Voltage (volts)	Current (Amp)	Power (Watts)	Energy (kWh) for 1 hr
Heater 1 (nearest to pully)	232	1.07	245.76	0.122
Heater 2 (middle)	230	1.03	227.424	0.133
Heater 3 (nearest to mold)	229	1.10	219.84	0.109

The motor used here consumes 1.1kWhr energy if it has been used for an hour. System total energy consumption becomes 1.464 kWhr.

A quantitative assessment was conducted to assess the machine's performance, involving multiple trials to produce yarn samples. The duration of operation and the quantity of raw material input remained consistent across each trial. Specific amounts of pellets were used, and the mass of the yarn output was measured. Three trials were conducted at varying speeds of motor shaft, and the findings are detailed in Table 6.

**Table 6:** Efficiency comparison for machine under varying motor speed.

screw speed (rpm	Input mass (in gram)	Output mass (in gram)	Average output (in gram)	Efficiency (%)
35	235	178.11	167.75	71.38 %
		155.25		
		169.90		
28.7	250	195.34	198.26	79.30 %
		210.55		
		188.89		
19.8	187	137.62	143.11	76.51 %
		142.58		
		149.12		

The findings suggest that the machine achieves its highest efficiency at 28.7 rpm. Across a range of motor speeds from 19 rpm to 35 rpm, the average efficiency of the machine is 75 %. This approach can be considered deemed efficient for a medium-scale production process that directly converts waste plastic into a product. Additional assessments of the machine's performance is factored into various independent process variables, such as screw speed (S), time (t), and power (P). Table 7 displays the dependent variables or responses of the machine, including recyclability (R), throughput (TP), and SME, calculated using the following formulas:

$$Recyclability\;\left(R\right)=\frac{Output\;mass\;of\;yarn\;\left(Q\right)}{Input\;mass\;of\;plastic\;flakes\;\left(I\right)}\times Throughput\left(TP\right)$$

$$Throughput\;\left(TP\right)=\frac{Output\;mass\;of\;yarn\;\left(Q\right)}{Time\;taken\;for\;recycling\;\left(t\right)}$$

$$Specific\;mechanical\;energy\;\left(SME\right)=\frac{Power\;\left(P\right)\times Time\left(t\right)}{Output\;mass\;of\;yarn\;\left(Q\right)}$$

$$Torque\;\left(T\right)=\frac{60\;\times Power\;\left(P\right)}{2\;\times \pi \times Screw\;speed\;\left(S\right)}$$

**Table 7:** Performance test for the machine (Effect of process variables on the machine responses).

S/N	Input (I) gm	Process Variables			Responses				
		Screw Speed (S) rpm	Power (W) watts	Time (t) min	Output (Q) gm	Torque (T) Nm	SME $\frac{\text{kJ}}{\text{kg}}$	Recyclability (R)%	Throughput (TP) $\frac{\text{gm}}{\text{hr}}$
1	200	27	1700	58	161.88	601.25	36.55	80.94	167.46
2	200	28.7	1645	67	152.27	547.34	43.43	76.135	154.85
3	200	32.6	1683	60	145.12	492.99	41.75	72.56	140.44
4	200	27.8	1700	62	188.89	583.95	33.48	94.445	188.89
5	200	33	1660	60	132.36	480.36	45.159	66.18	132.36

**Fig. 23:**
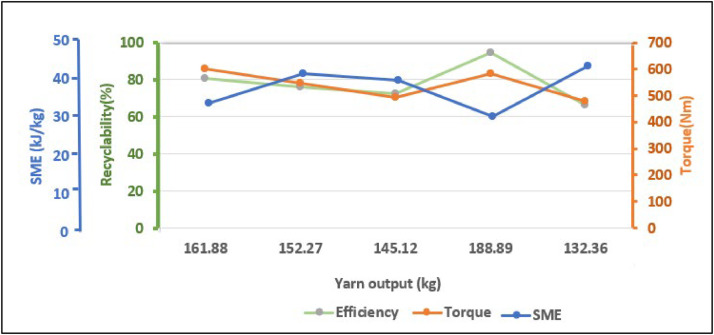
Effect of torque and SME on the recyclability and yarn production.

The data from Table 7 is illustrated in Fig. 23, signifying that the machine achieves its highest output, producing 188.89 g of yarn at a maximum efficiency of 94 %, when the screw speed is lower, but the torque is higher. This setup leads to minimal SME, therefore decreasing operational expenses and energy usage. The average throughput of this machine is 156.8 g per hour.

The results of this experiment can be summarized that a reduction in screw speed leads to an increase in torque, while keeping the temperature zones constant inside the barrel. This leads to enhance production, improved recyclability, increased throughput, and reduced SME.

## Conclusion and further improvements

Advancement of thread construction through extrusion has significant potential as a highly promising technology, offering ample opportunities for further enhancement and innovation. The aim of this project is to create thread using recycled waste plastic bottle. This project introduces a more compact and small-scale machine to produce thread. Also, this proposed device is completely detachable to improve transportability whereas the other extruder machines are comparatively large scale and cannot be moved at any time easily. The optimum output is obtained when the temperature ranges from 170 °C to 211 °C. The efficiency of the device can be increased by decreasing the heat conduction and incorporating proper temperature insulation. The experiment results showed that at lower screw speed around 27.8 rpm can elevate torque, while maintaining consistent temperature zones within the barrel. Thus, this leads to heightened production, enhanced efficiency, increased throughput, and lowered SME. A decrease in screw speed will also enable the utilization of PVC powder for future applications, therefore requiring less heat to achieve equivalent mixing levels. This will also save material cost, as PVC powder blends are cheaper than pellets. The machine can become more efficient by incorporating additional motors, iron cylinders, and smart cooling mechanisms. The project's constraint stems from the disparity in diameter between the barrel and screw, resulting in an accumulation of excess plastic material within the barrel. As this material cools down, it solidifies, reducing the efficiency of the system. In this context, it is hard to conclude the effectiveness of this system, but the outcome of the proposed system is promising, which leaves the scope to forward the work for improvements.

## CRediT authorship contribution statement

**Nuzat Nuary Alam:** Supervision, Conceptualization, Methodology, Visualization, Writing – original draft, Writing – review & editing. **Md. Mehrab Sadik:** Validation, Investigation, Project administration. **Tahmid Shahriar Arnob:** Validation, Investigation, Project administration. **Isfak Habib Iftu:** Software, Resources. **Abrar Jahin Khan:** Software, Funding acquisition. **Kazi Firoz Ahmed:** Writing – review & editing. **Rethwan Faiz:** Conceptualization, Writing – review & editing.

## Declaration of competing interest

The authors declare that they have no known competing financial interests or personal relationships that could have appeared to influence the work reported in this paper.

## Data Availability

Data will be made available on request.
